# Multiband Envelope Spectra Extraction for Fault Diagnosis of Rolling Element Bearings

**DOI:** 10.3390/s18051466

**Published:** 2018-05-08

**Authors:** Jie Duan, Tielin Shi, Hongdi Zhou, Jianping Xuan, Yongxiang Zhang

**Affiliations:** 1 State Key Lab of Digital Manufacturing Equipment and Technology, Huazhong University of Science and Technology, Wuhan 430074, China; jduan@hust.edu.cn (J.D.); jpxuan@hust.edu.cn (J.X.); 2 School of Mechanical Engineering, Hubei University of Technology, Wuhan 430068, China; zh_hongdi@hust.edu.cn; 3 Department of Power Engineering, Naval University of Engineering, Wuhan 430033, China; zyx6302@aliyun.com

**Keywords:** narrowband amplitude demodulation, Jarque-Bera statistic, rolling element bearing, blind source separation, fault diagnosis

## Abstract

Bearing fault features are presented as repetitive transient impulses in vibration signals. Narrowband demodulation methods have been widely used to extract the repetitive transients in bearing fault diagnosis, for which the key factor is to accurately locate the optimal band. A multitude of criteria have been constructed to determine the optimal frequency band for demodulation. However, these criteria can only describe the strength of transient impulses, and cannot differentiate fault-related impulses and interference impulses that are cyclically generated in the signals. Additionally, these criteria are easily affected by the independent transitions and background noise in industrial locales. Therefore, the large values of the criteria may not appear in the optimal frequency band. To overcome these problems, a new method, referred to as multiband envelope spectra extraction (MESE), is proposed in this paper, which can extract all repetitive transient features in the signals. The novelty of MESE lies in the following aspects: (1) it can fuse envelope spectra at multiple narrow bands. The potential bands are selected based on Jarque-Bera statistics of narrowband envelope spectra; and (2) fast independent component analysis (fastICA) is introduced to extract the independent source spectra, which are fault- or interference-related. The multi-band strategy will preserve all impulse features and make the method more robust. Meanwhile, as a blind source separation technique, the fastICA can suppress some in-band noise and make the diagnosis more accurate. Several simulated and experimental signals are used to validate the efficiency of the proposed method. The results show that MESE is effective for enhanced fault diagnosis of rolling element bearings. Bearing faults can be detected even in a harsh environment.

## 1. Introduction

Rolling element bearings (REB) are widely used in rotating machinery. Unexpected faults occurring in bearings may lead to fatal breakdown of machines and result in enormous economic losses in industry. Therefore, it is important to accurately detect defects in bearings early. Vibration signals are widely used for bearing fault diagnosis [1,2,3]. When a rolling element moves over a damaged area, such as crack occurring on a bearing component, a shock impulse will be generated. These impulses excite the resonance of the bearing or adjacent components at a special rate called “bearing characteristic frequencies” [4]. The characteristic frequencies are governed by the operating speed of the unit and the geometry of the bearing, which can indicate the status and fault types of REB. Hence, the key issue for bearing fault diagnosis is the detection of the characteristic frequencies [5].

Envelope analysis is able to highlight the fault characteristic frequencies of vibration signals, which have been widely used in bearing fault diagnosis [6]. Since fault features are extremely weak and are often seriously contaminated by noise in some industrial cases, or by the early stages of damage, envelope analysis cannot meet the diagnostic requirements of fault detection. Hence, numerous techniques have been proposed to decompose the signal before envelope analysis to improve the signal-to-noise ratio (SNR). In [7], a bearing fault diagnosis method was proposed by combining wavelet transform with envelope analysis. Tse et al. [8] proposed an innovative wavelet transform called exact wavelet analysis. At each selected time frame, the method generates an adaptive daughter wavelet to match the inspected signal as exactly as possible. In [9], the authors used a Morlet wavelet to eliminate the frequency associated with interferential vibrations, and the autocorrelation envelope spectrum was applied to highlight the periodic impulsive features. A hybrid signal-processing method [10] based on ensemble empirical mode decomposition (EEMD) has been proposed. The raw vibration signal of faulty bearings is filtered by an optimal band-pass filter, and then EEMD is applied to decompose the filtered signal. All of the methods mentioned above are focused on signal decomposition in the temporal domain and then extract the fault-related sub-signals for envelope analysis.

The problem of fault feature extraction can also be solved by the narrowband amplitude demodulation method, which is a combination of band-pass filter and envelope analysis. Narrowband demodulation methods offer, strong and reliable diagnostic potential, since they are based on a solid physical background, which can greatly improve the SNR. There are two key issues in narrowband demodulation: the design of the filter to segment the signal, and the construction of criteria with which to determine the informative frequency band. The idea of applying kurtosis for sub-signals introduced by Antoni [11] has captured much attention. Antoni combined the short-time Fourier transform (STFT) and spectral kurtosis (SK) [12] to construct a Kurtogram that could be used to discover the optimal frequency band for the diagnosis of a rotating machinery fault. To reduce computing time, a fast Kurtogram estimator [13] was also proposed, which is a potential tool for online industrial applications. Wang and Liang [14,15] proposed an adaptive SK technique that could optimize filter bandwidth and locate the center frequency for fault detection of rotating machinery. This technique is implemented by right-expanding an initial window along the frequency axis via successive attempts to merge it with its subsequent neighboring windows. In [16], the frequency spectrum of the bearing signal was firstly divided into fine initial segments, which were then adaptively merged into different subsets using an enhanced bottom-up segmentation technique. Three commonly used criteria, i.e., kurtosis, smoothness index (SI), and crest factor (CF), are fused into a combined criterion based on the entropy principle to guide the spectral segmentation and merging process. In all these methods above, the criteria for band selection are calculated based on the filtered time signal.

Since kurtosis of temporal signals can be considerably affected by noise and large random impulses, Barszcz and Jabłoński [17] recently raised the idea of constructing criteria based on envelope spectrum amplitudes. They pointed out that the fast Kurtogram did not yield any significant band in machinery fault detection when the SNR was relatively low or the signal contained a variety of peaks, while the Protrugram technique, based on kurtosis of the envelope spectrum amplitudes of narrowband signals, could work well. The effectiveness of the idea of constructing criteria based on the envelope spectrum has been validated by other researchers, as well. Wang et al. [18] proposed an enhanced Kurtogram by calculating kurtosis based on the power envelope spectra of the signals extracted from wavelet packet nodes at different depths, which performed well in determining the location of the resonant frequency band. Antoni et al. [19] defined the squared envelope infogram (SE infogram) and squared envelope spectrum infogram (SES infogram) by measuring negentropy of the squared envelope (SE) and squared envelope spectrum (SES) of the signal, respectively. The average of SE infogram and SES infogram can detect repetitive transients, even in the presence of strong impulsive noise. Tse and Wang [20] constructed a sparsogram using sparsity values of power spectra for the envelope of wavelet packet coefficients. The sparsogram can effectively detect bearings with different types of faults, as well as multiple faults. To solve the problem of the bearing’s resonant frequency band being located in the overlapping frequency band of a wavelet filter bank, they also presented an automatic band-selection method [21] for finding the optimal complex Morlet wavelet filter with the help of the genetic algorithm to maximize the sparsity measurement value. In [22], the authors proposed an improved correlated kurtosis on the basis of SES to detect repetitive transient impulses. The method based on this criterion, combining wavelet filter and the Particle Swarm Optimization (PSO) algorithm, can adaptively determine the optimal resonant frequency band and accurately demodulate transient fault features of rolling element bearings.

Criteria constructed on the envelope spectrum are efficient for discovering the periodic repetitive impulses in a signal. However, it is hard to determine the fault-related resonance band via the values alone, since the criteria are particularly vulnerable to the impact of casual impulses. For example, periodically generated interference impulses are also converted to impulses in the envelope spectrum and lead to a large criterion value. In circumstances with heavy interferences, large criterion values can appear in several frequency bands, and the largest criterion value may not appear in the optimal band. Hence, selecting a signal in only one frequency band may leave out quite a few transient features, and a multi-band strategy is more robust since it can retain all potential bands. Considering the merits of criteria calculated using the envelope spectrum and multi-band strategy, a novel method named multiband envelope spectra extraction (MESE) is proposed in this paper. The proposed method can extract all narrowband envelope spectra with repetitive impulse components in the signal, which can be used to detect characteristic frequencies of the faulty bearing. Simulation and experimental results demonstrate that the proposed method is more accurate and robust than the narrowband demodulation methods in bearing fault diagnosis.

The remainder of this paper is arranged as follows. Section 2 describes how to extract multiple impulse-related envelope spectra for bearing fault diagnosis. The band selection technique based on Jarque-Bera (*JB*) statistics is also described in this section. Simulation analysis of the proposed method is discussed in Section 3. Then, in Section 4, some real data obtained in the laboratory are used to validate the effectiveness of the proposed method. The fast Kurtogram and the Protrugram are also conducted to analyze the same signals for comparisons. Finally, conclusions are provided in Section 5.

## 2. Multiband Envelope Spectra Extraction

The proposed method is divided into several steps in this section. Firstly, the frequency band segmentation process is introduced. Then, a band determination technique based on the *JB* statistics is proposed. Finally, envelope spectra at multiple bands are selected, and fast independent component analysis (fastICA) is introduced to extract the impulsive source spectra in these narrow bands. The details are described in the following subsections.

### 2.1. Frequency Band Segmentation

**Figure 1:**
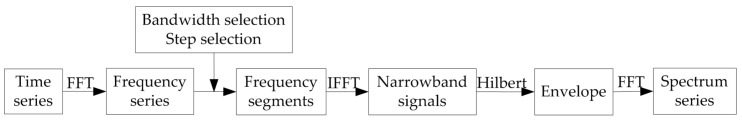
Flowchart of frequency band segmentation.

The signal is split into segments in the frequency domain, and the criterion based on envelope spectrum is calculated. The band segmentation process can be explained as a rectangular window with the length of the bandwidth truncating the frequency series. Central frequencies of the window that shift over the frequency range are determined by the bandwidth and shift step, which are the two most crucial parameters in this technique. It is a simple but effective way to intercept the frequency band and to obtain the narrowband signals. The flowchart is depicted in Figure 1. Details of the process are introduced in the following.

Suppose the acquired vibration signal is a time series *x*(*k*) (where *k* = 0, 1, …, *N* − 1), *N* is the length of the discrete series. In the segmentation process, the signal is first transformed to the frequency domain by discrete Fourier transform (DFT) [14,23].

(1) $$X(n)=\sum_{k=0}^{N-1}x(k)e^{-j(2\pi /N)kn},$$

where *X* are Fourier coefficients of the signal. Then a rectangular window is defined to truncate the frequency series:

(2) $$w(n)=\{\begin{array}{cc} 1 & 0\leq n<BW\\ 0 & else \end{array},$$

where *BW* is the length of the window. Frequency band segmentation is realized by slipping the window over the frequency series.

(3) $$X_{i}(n)=X(n)\cdot w(n-(i-1)a),$$

*X_i_* are Fourier coefficients of the *i*th segment and *a* is the shift step length or iteration step length. Then, the frequency segment series *SX* can be expressed as

(4) $$SX=\left\{X_{1},X_{2},...,X_{m}\right\},$$

where *m* is the number of segments, which is determined by the length of the discrete series *N*, window length *BW* and shift step length *a*.

(5) m=⌊(N/2−BW)/a⌋.

The symbol ⌊ ⌋ in Equation (5) means to get the maximum integer that is not more than the operated data. The shift step length *a* and window length *BW* have a significant influence on the determination of *SX*, which should be carefully picked.

After the frequency segment series *SX* is obtained, the criterion of the envelope spectrum amplitudes in each narrowband should be calculated. Each frequency segment is firstly transformed into the time domain by inverse Fourier transform. The envelope of the sub-signal in each narrow band can be obtained by Hilbert transform (HT) [6,24]. Then, narrowband envelope spectrum *X_i_^e^* is obtained through fast Fourier transform (FFT) of the envelope. Thus, the acquired envelope spectrum series can be expressed as

(6) $$X^{e}=\left\{{X_{1}}^{e},{X_{2}}^{e},...,{X_{m}}^{e}\right\}.$$

Periodic repetitive transient impulses in the time signal can bring impulses in to the envelope spectrum. Therefore, the next step is to identify these narrow bands with impulsive envelope spectra.

### 2.2. Narrow Band Selection

Kurtosis of the envelope spectrum can well reflect the impulsiveness of signal. For the *i*th narrow band, kurtosis [17] of its envelope spectrum *X_i_^e^* is defined as

(7) $$K_{i}=\frac{(1/N)\sum_{j=0}^{N-1}{\left[{X_{i}}^{e}(j)-((1/N)\sum_{j=0}^{N-1}{X_{i}}^{e}(j))\right]}^{4}}{{\left\{(1/N)\sum_{j=0}^{N-1}{\left[{X_{i}}^{e}(j)-((1/N)\sum_{j=0}^{N-1}{X_{i}}^{e}(j))\right]}^{2}\right\}}^{2}}.$$

In the existing methods [13,14,15,17,18], the band with maximum or protruding kurtosis is designated as the optimal band. However, the repetitive interference impulses can also bring impulses in to the envelope spectrum, which can complicate the diagram of kurtosis. Therefore, it is difficult to determine the right band only via the protruding values. In addition, in-band noises can weaken the visual inspection ability of extracted narrowband envelope spectrum.

**Figure 2:**
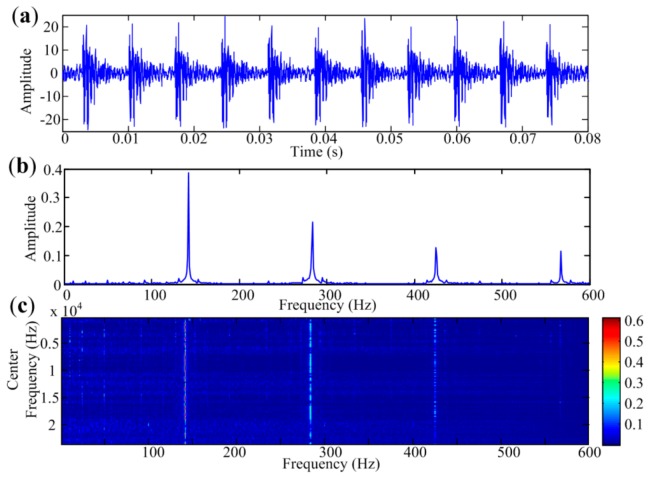
REB fault signal with small noises. (**a**) Temporal waveform; (**b**) Traditional envelope spectrum of the signal; (**c**) Envelope spectra at different narrow frequency bands.

In bearing vibration signals, fault-related features are presented as repetitive transient impulses. Since energies of impulses in the time signal spread over a wide frequency range, bearing fault features may be contained in narrowband signals with different central frequencies, which are presented as impulses in the envelope spectra. To verify the characteristics of repetitive impulses presented in the bearing fault signal, a real fault signal with small noise is analyzed in this section. Figure 2a is the temporal waveform of the signal and Figure 2b is its envelope spectrum obtained by traditional envelope analysis. Normalized envelope spectra of the narrowband signal with different center frequencies are paved in Figure 2c. It can be seen that the first three harmonics of the bearing fault frequency are visible in the spectra of signals in different narrow bands. The shape of envelope spectrum at each narrow band is the same as that of the traditional envelope spectrum, as shown in Figure 2b, which means the envelope spectra of periodic transient impulses have inherent structure. Meanwhile, background noise is randomly distributed among the envelope spectra at selected narrow bands. So multi-band strategy is feasible for extracting all impulse features and suppressing the in-band noises. Since multiple bands are selected, not only should the impulses in the narrowband envelope spectrum be detected, but also the bands with protruding impulses should be well differentiated from those dominated by noises. We will use the *JB* statistic of the envelope spectrum to determine the candidate bands as it performs better than kurtosis in the detection of impulsiveness [25].

The *JB* statistic is a combination of empirical skewness and kurtosis, and has been used to test whether sample data match a Gaussian distribution. For the *i*th narrow band, the *JB* statistic [25] of the envelope spectrum *X_i_^e^* is defined as

(8) $$JB_{i}=\frac{N}{6}\left[{S_{i}}^{2}+\frac{{\left(K_{i}-3\right)}^{2}}{4}\right],$$

where *S_i_* is skewness of the envelope spectrum *X_i_^e^* and can be calculated as

(9) Si=(1/N)∑j=0N−1[Xie(j)−((1/N)∑j=0N−1Xie(j))]3{(1/N)∑j=0N−1[Xie(j)−((1/N)∑j=0N−1Xie(j))]2}32.

**Figure 3:**
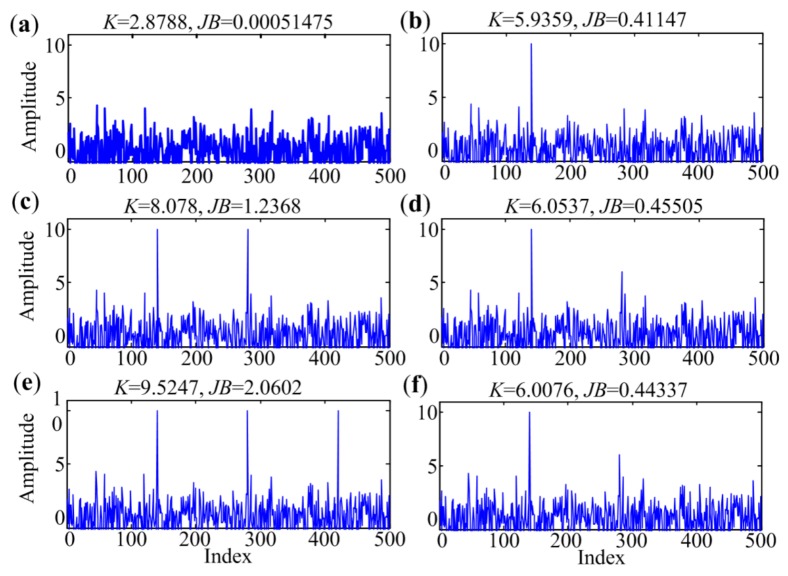
Kurtosis and *JB* statistics of narrowband envelope spectra with possible shapes: (**a**) Without harmonics; (**b**) One harmonic; (**c**) Two harmonics having the same amplitudes; (**d**) Two harmonics having descending amplitudes; (**e**) Three harmonics having the same amplitudes; (**f**) Three harmonics having descending amplitudes.

In order to evaluate the performance of *JB* statistic in band determination, a few simulated envelope spectra are generated, as per [17]. The harmonics of repetitive transients in the narrowband spectrum are considered to be impulses with regular intervals in the simulation. Figure 3a is the envelope spectrum without any fault features, and b–f show possible shapes of narrowband envelope spectra illustrating the presence of periodic impulses. Some Gaussian noises are added to the pure spectra to simulate real conditions. The *JB* statistic (*JB* in the figure) and kurtosis (*K* in the figure) are calculated for comparison and presented at the top of each sub-figure. As can be seen in the figure, the value of the *JB* statistic varies dramatically when harmonics are present in the spectrum. However, for the spectra with or without harmonics, the values of kurtosis change very little, since they have the same order of magnitude. Hence, *JB* statistic is more sensitive than kurtosis in harmonics detection in the narrowband envelope spectrum, which can make the criteria of these bands with impulse features more protruding in the criterion series. Since the threshold for separating informative bands and bands dominated by noise is defined based on all of these values of the criterion, the protrusion can make the process more accurate.

After calculating the *JB* statistic of every narrowband envelope spectrum, the criterion series is obtained as *JB_i_* (where *i* = 1, 2, …, *m*). Since values of *JB* statistic can be indicative of the impulsiveness of a narrowband signal, the mean of the *JB* statistic series expressed as *JBT* is calculated as the threshold. This threshold is used to filter out those bands whose envelope spectra are drowned in noise.

(10) $$JBT=(1/m)\sum_{i=1}^{m}JB_{i}.$$

The envelope spectra of these narrow bands with *JB* statistics bigger than *JBT* are selected to form the informative spectra series *Y* = {*Y*_1_, *Y*_2_, …, *Y_h_*} (where *Y_i_* is envelope spectrum of the *i*th selected band). All these narrow bands are potential bands where transient impulses occur, but in which the envelope spectra may also be masked by noise.

### 2.3. Blind Source Separation

As described in the last subsection, the bands with criteria bigger than the threshold have envelope spectra containing impulses, which correspond to repetitive transient impulses cyclically generated in the time signal. These impulses are caused by a bearing fault or interference that can be distinguished by frequency components in the spectra. The informative spectra may contain fault-related features, inference-related features, or both. Therefore, each selected spectrum is a combination of a fault-related spectrum and an interference-related spectrum. The pure fault spectrum and interference spectrum both have definite shapes that contain impulses with regular intervals. In the simplest form, the selected spectra *Y*_1_, *Y*_2_, …, *Y_h_* are assumed to be linear combinations of *l* unknown independent spectra *E*_1_, *E*_2_, …, *E_l_* (*l* ≤ *h*). *E_i_* is the *i*th pure source envelope spectrum related to repetitive transient impulses. Let us arrange the spectra *Y_i_* into a vector *Y* = (*Y*_1_, *Y*_2_, …, *Y_h_*)^T^ and the source spectra into another vector *E* = (*E*_1_, *E*_2_, …, *E_l_*)^T^, respectively. Then, the linear relationship is given by

(11) $$Y=AE+N_{t},$$

where *A* is an unknown *h* × *l* matrix, which is referred to as a mixing matrix, and *N_t_* is random noise. The basic problem is to extract the source spectra *E* from the selected spectra *Y* or, equivalently, to estimate the mixing matrix *A*. This is a blind source separation (BSS) problem.

There are several algorithms to solve BSS problems, such as Jutten-Hérault algorithm [26], nonlinear principle component analysis (PCA) [27], independent component analysis (ICA) [28], the information maximization approach [29], equivariant adaptive separation via independence (EASI) [30], fastICA [31], etc. In this paper, the fastICA is introduced to reveal the source components from the selected narrowband envelope spectra. The fastICA is exceedingly simple, does not depend on any user-defined parameters, and is quick to converge to the most accurate solution allowed by the data.

The fastICA is an improved method of ICA, and has the same principle as ICA. ICA of the vector *Y* consists of finding a linear transform matrix *W* to make the components *E* = *W^T^Y* as independent as possible, in the sense of maximizing some function that measures independence. ICA is usually performed by formulating an objective function, which is also called a contract function, and then minimizing or maximizing it. The two key factors for fastICA or ICA are source number estimation and the definition of contrast function, which will be discussed next. The following gives a succinct description for ICA and fastICA used in our method.

In this study, the ICA problem is solved by negentropy maximization. Negentropy can be interpreted as a measure of nongaussianity. For a vector *y* = (*y*_1_, *y*_2_, …, *y_n_*)*^T^*, negentropy [28] is defined as follows:

(12) $$J(y)=H(y_{gauss})-H(y),$$

where *y_gauss_* is a Gaussian random vector that has the same covariance matrix as *y*. *H*(*.*) is the differential entropy of the vector and is defined as

(13) $$H(y)=-\int p_{y}(\xi )\log p_{y}(\xi )d\xi ,$$

where *p_y_*(*.*) is the vector’s possibility density. Negentropy is always non-negative, and is zero if and only if *y* has a Gaussian distribution. Finding a transformation matrix to extract source data is roughly equivalent to finding the projection directions in which the negentropy is maximized. It is impossible to get the possibility density of the vector in real cases. Therefore, a simple estimate of the negentropy is needed. Many approximations have been developed [32] based on the maximum entropy principle. One of them has the form:

(14) $$J(y)\approx c{\left[E\left\{g(y)\right\}-E\left\{g(v)\right\}\right]}^{2},$$

where *c* is a constant, *v* is a Gaussian variable of zero mean and unit variance, and *g* is a nonquadratic function. In this study, the function is defined as

(15) $$g(u)=\frac{1}{b}\log\cosh(bu),$$

where 1 ≤ *b* ≤ 2 is constant. The approximation formula can give a new objective function to estimate the ICA transform. To find one source spectrum *E_i_* = *W_i_^T^Y* in this study, the projection direction can be obtained by maximizing the function *J_g_* given by

(16) $$J_{g}(W_{i})={\left[E\left\{g({W_{i}}^{T}Y)\right\}-E\left\{g(v)\right\}\right]}^{2}.$$

As an optimization process, the independent components can be estimated one by one. In fastICA, the fixed-point iteration algorithm that computes in batch mode is introduced to maximize the objective function and makes the convergence fast and reliable. After some reasonable approximations, it can obtain the following iteration [32]:

(17) $${W_{i}}^{+}=E\left[Yg({W_{i}}^{T}Y)\right]-E\left[g^{\prime }({W_{i}}^{T}Y)\right]W_{i},\;{W_{i}}^{*}={W_{i}}^{+}/\left\|{W_{i}}^{+}\right\|,$$

where W_i_^*^ denotes the new value of *W_i_*. The iteration can be used to optimize the matrix *W* and separate these independent source spectra.

In this study, the extraction of source envelope spectra is an overcomplete ICA problem, such as *h* ≥ l. Therefore, the number of source spectra in the signal needs to be accurately estimated. As these observed vectors will formulate a high dimension matrix, it is a dimension reduction process and can be realized by a PCA-related method. The main step in PCA is to calculate the covariance matrix of the original data and find out the eigenvectors and eigenvalues of the matrix. The problem of detecting the number of sources is equivalent to determining the number of source eigenvalues. The rearranged eigenvalues can be expressed as

(18) $$\lambda_{1}\geq \lambda_{2}\geq \cdot \cdot \cdot \geq \lambda_{l}\geq \lambda_{l+1}\geq \cdot \cdot \cdot \geq \lambda_{h}.$$

There are many kinds of indexes [33,34,35,36] for determining the number of principal components. Here we consider the differences between adjacent eigenvalues [33] in Equation (18), which lead to the criteria:

(19) $$d_{i}=\lambda_{i}-\lambda_{i+1},i=1,2,...,h-1.$$

The shapes of source spectra are different from each other, while the same source spectrum appeared in multiple narrow bands often has definite shape. The difference *d_i_* will be small when *λ_i_* and *λ_i_*_+1_ are both noise eigenvalues, but large if one or both are source eigenvalues. This suggests that estimating the number *l* is equivalent to estimating the multiplicity of the eigenvalues. The test starts checking the difference of adjacent eigenvalues from *i* = 1. When the difference is less than the alarm threshold *ε*, the detection scheme is over, which corresponds to the interface between the signal and noise subspaces. As a result, we get the ideal source number estimation.

After getting the number of independent source spectra and defining the contrast function, the main steps of fastICA are given as follows:

**Step 1:** Center and whiten the measured data *Y*;

**Step 2:** Estimate the number of source *l* in the system based on differences of adjacent eigenvalues and let *p* = 1;

**Step 3:** Choose a random initial vector *W_P_* with norm 1 and determine the loop’s termination condition;

**Step 4:** Let $W_{p}=E\left[Yg({W_{p}}^{T}Y)\right]-E\left[g^{\prime }({W_{p}}^{T}Y)\right]W_{p}$, where *g*(**.**) is the nonquadratic function as Equation (15) and *g′*(**.**) is the derivative of *g*(**.**);

**Step 5:** Perform Gram-Schmidt orthogonalization to achieve decorrelation:

$$W_{p}=W_{p}-\sum_{j=0}^{p-1}\left({W_{p}}^{T}W_{j}\right)W_{j}\;W_{p}=W_{p}/\left\|W_{p}\right\|$$

**Step 6:** If the iteration is not convergent, go back to step 4;

**Step 7:** Let *p* = *p* + 1. If *p* ≤ *l*, go back to step 3. Otherwise, output the optimal matrix *W*, which is constructed by all the vectors *W_i_*.

### 2.4. Summary of the Proposed Method

**Figure 4:**
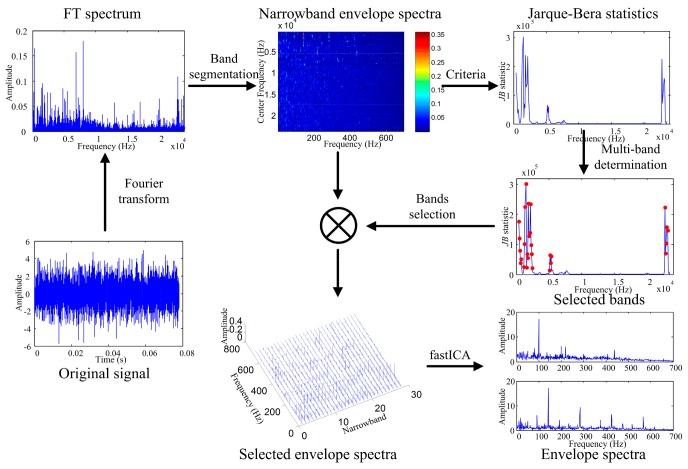
Flowchart of the proposed method for bearing fault diagnosis.

Based on the above discussions, the procedure of the proposed method for detecting REB faults can be briefly described in Figure 4. First, the measured vibration signal is transformed to the frequency domain. Then, a rectangular window with constant length is used to truncate the frequency band and obtain whole narrowband segments. Next, informative bands are determined based on *JB* statistics of narrowband envelope spectra, as introduced in Section 2.2. Mean value of *JB* statistics is calculated as the threshold. Envelope spectra of the selected narrow bands can be seen as observed data in BSS, and the source data are truly impulse-related spectra. Finally, the fastICA algorithm is used to extract the source envelope spectra, and the number of source spectra is determined by differences of adjacent eigenvalues. These source spectra contain features for bearing fault diagnosis.

## 3. Simulation

**Figure 5:**
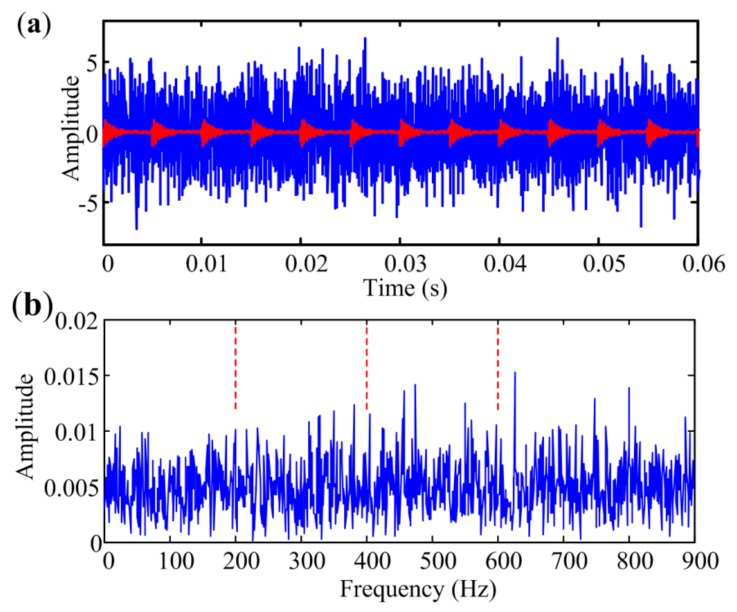
(**a**) Periodic impulses and the noise signal; (**b**) Envelope spectrum of the simulated fault signal.

In the simulation, the fault signal is modeled as a series of repeated damping oscillation waveforms [37]. It is an amplitude-modulated signal in which the fault characteristic frequency is modulated to a high resonance frequency carrier. The simulated signal *x*(*t*) can be computed by the following formula

(20) $$x(t)=\sum_{j=1}^{M}A_{j}s(t-jT)+n(t),$$

where *s*(*t*) is the oscillating waveform, *n*(*t*) is Gaussian white noise, *A_j_* is the amplitude of the *j*th impulse. *M* is the number of impulses and *T* represents the time period corresponding to the characteristic frequency. In the above equation, *s*(*t*) is defined as

(21) $$s(t)=e^{-\beta t}\cos(2\pi f_{r}t),$$

where *β* is the damping coefficient, *f_r_* is modulation frequency, which reflects the resonance information of the system. The value of *A_j_* is related to the location of the defect in rolling element bearings [37] and is simplified to be constant in the simulation. Sampling time is set to 1 s, and the other parameters mentioned above are given in Table 1. Hence, the characteristic frequency *f_c_* of the simulated bearing fault is 200 Hz. There will be about 65,000 samplings in the simulated signal, but only 4000 of them are shown in the figure to display clearly. The simulated signal and the pure impulses with red lines are presented in Figure 5a. The periodic transient impulses are contaminated by heavy noise. The envelope spectrum of the simulated signal obtained through HT and FFT is presented in Figure 5b. It can be seen that *f_c_* and its harmonics are not evident in the spectrum.

**Figure 6:**
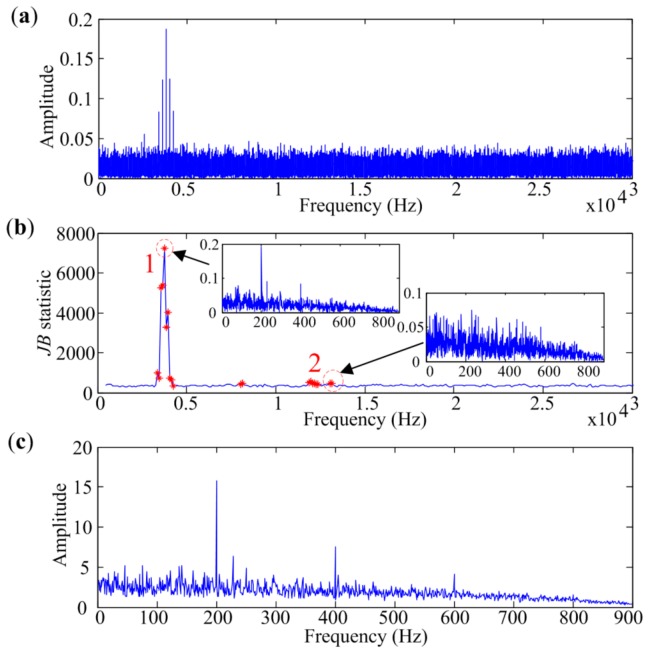
(**a**) Amplitude spectrum of the simulated signal; (**b**) *JB* statistics calculated at different bands; (**c**) Envelope spectrum extracted by MESE.

MESE is used to analyze the signal presented in Figure 5a, and the shift step size is set to 100 Hz. As it is often desirable to detect up to the third harmonic of the bearing defect frequency in the envelope spectrum for fault diagnosis [38], the bandwidth should be more than three times the sought characteristic frequency. However, the harmonic near the cut-off frequency is easy to overwhelm with noise in narrowband envelope spectrum calculation. Therefore, the bandwidth in MESE is set to 900 Hz, slightly bigger than four times the characteristic frequency in the simulation. The result is illustrated in Figure 6. Figure 6a shows the amplitude spectrum of the simulated signal. *JB* statistics of narrowband envelope spectra are presented in Figure 6b. Potential envelope spectra at different bands that are marked with red asterisk in Figure 6b are selected for further analysis. The acquired envelope spectrum is shown in Figure 6c, which shows that the first three harmonics of *f_c_* are quite effectively extracted. Sub-figures in Figure 6b also present the envelope spectra extracted by narrowband demodulation at two selected narrow bands. Although the characteristic frequency can be identified by narrowband envelope spectrum with the biggest criterion, the third harmonic is overwhelmed by in-band noise. It also can be seen that no characteristic component appears in the narrowband spectrum selected at point 2 in Figure 6b, but has little influence on the performance of MESE. Therefore, MESE is effective and robust in bearing fault diagnosis.

**Figure 7:**
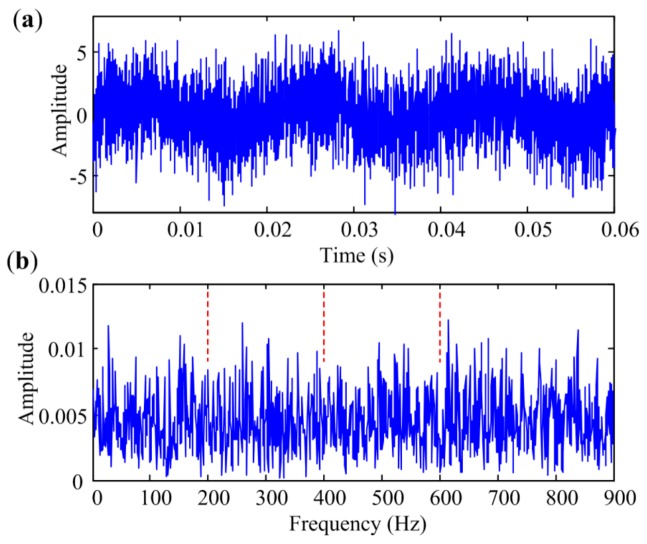
(**a**) Simulated signal with heavy interferences; (**b**) Envelope spectrum of the signal.

In industrial locales, quite a few other repetitive transient impulses periodically excited by mechanical elements may also appear in the signal, and these comprise the interference impulse components. Meanwhile, sinusoids brought in by the shaft machine are familiar in vibration signal. Hence, we also simulate the REB fault signal with these two interferences. The impulse interference can be easily realized by adding another impact signal with resonance frequency *f_r_*_1_ (6000 Hz) and characteristic frequency *f_c_*_1_ (260 Hz) to the simulated signal above. Rotational frequency *f_R_* of the sinusoid is 50 Hz, and the amplitude is 1.0 in this case. The simulated signal and its envelope spectrum are presented in Figure 7. The characteristic frequency and its harmonics are swallowed by these interferences in the envelope spectrum as shown in Figure 7b.

**Figure 8:**
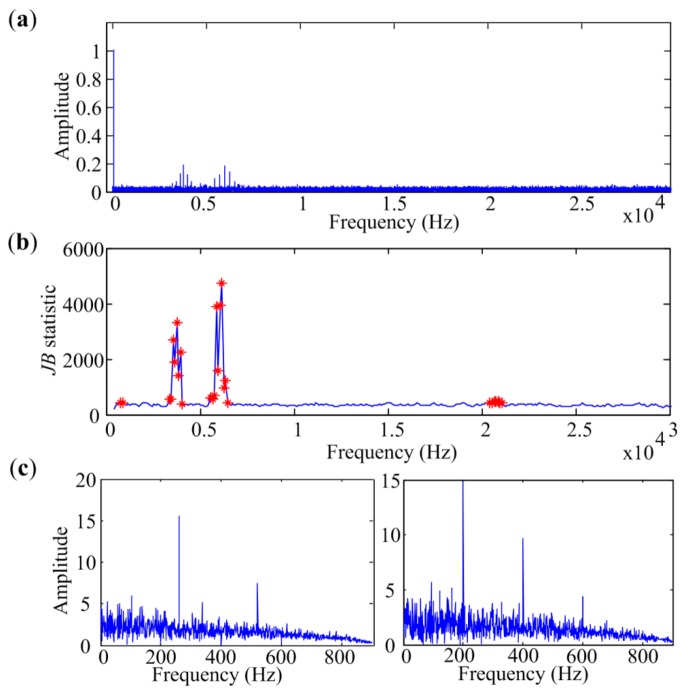
Analyzed results of signals with heavy interferences: (**a**) Amplitude spectrum; (**b**) JB statistic diagram; (**c**) Envelope spectra extracted by MESE.

Figure 8a presents the amplitude spectrum of the simulated signal and the diagram of *JB* statistic is displayed in Figure 8b. It can be seen that there may be two bands containing the impact components, and the band with central frequency 6160 Hz has the biggest *JB* statistic. If only the band with the biggest criterion is selected for further analysis, the fault-related features will not be detected. The criterion diagram can be more complicated in real cases, which need more manual intervention to choose the right frequency band for demodulation and will complicate the fault diagnosis. In MESE, multiple discrete narrow bands are selected for further analysis. Figure 8c shows the final result obtained by MESE, and two independent source spectra are extracted. The right one is a fault-related spectrum, in which the fault frequency, 200 Hz, and its harmonics are clearly visible. And the envelope spectrum of the interference component 260 Hz is also extracted corresponding to the left figure. The sinusoid is wiped off in both spectra. The results demonstrate that MESE can effectively detect bearing faults even in the low signal-to-noise situation with heavy interferences.

## 4. Experiment

**Figure 9:**
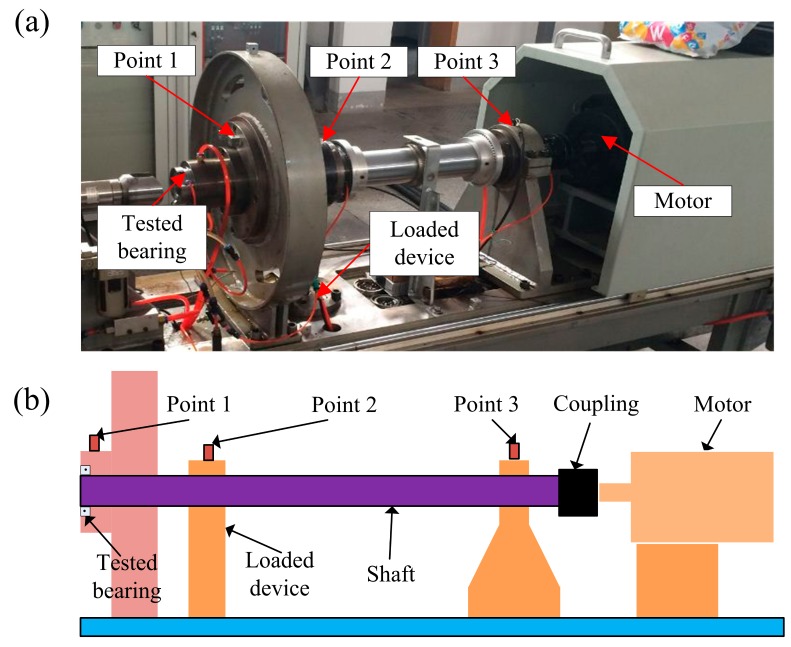
Bearing test rig and sensor placement illustration.

To verify the effectiveness of the proposed method in practical applications, we established a simplified bearing test rig, which is shown in Figure 9a. In order to explain the structure clearly, a sketch of the test rig is presented in Figure 9b. The test rig is made up of an induction motor, a shaft coupling to the motor, several bearings supporting the shaft, and an oil-loaded device as a radial loader. Tested bearings are installed at the left end of the shaft.

It may be impossible to mount sensors near the failure bearings in quite a few situations of industrial field, so fault diagnosis by signals acquired at long distance is meaningful in practical applications. In the experiment, we simulated situations in which the measuring points were far away from the defective bearing. Three accelerometers were mounted along the shaft at Point 1, Point 2 and Point 3 to acquire vibration signals, as shown in Figure 9a. These signals obtained away from the tested bearing are often severely tainted by various noises and interfering vibrations caused by other machine elements. Consequently, it will be difficult to detect the bearing faults in these situations.

The type of tested bearing is HRB 6010-2RZ (Harbin Bearing Manufacturing Co., Ltd., Harbin, China), and parameters of the bearings are listed in Table 2. Spalls were introduced by wire-cut electro-discharge machining (WEDM) on the inner race and the outer race, respectively. Rotation speed was kept constant at 1500 rpm in the experiment. A radial load of 2.0 kN was added to the shaft and bearings. Sampling frequency of the data acquisition system is 65,536 Hz.

### 4.1. Bearing Inner-Race Defect Identification

**Figure 10:**
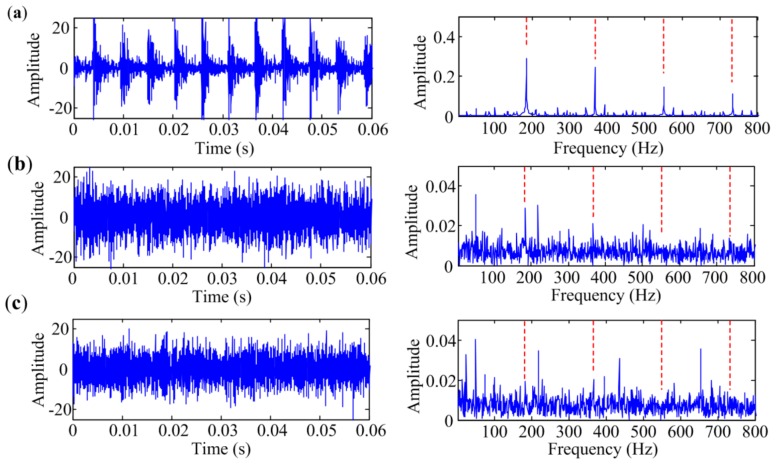
Bearing vibration signals measured at different points with inner-race defect: (**a**) Point 1; (**b**) Point 2; (**c**) Point 3.

As rotation speed is kept constant at 1500 rpm, the characteristic frequency of inner-race defect *f_I_* is 185 Hz in this condition. The temporal waveforms and corresponding envelope spectra are illustrated in Figure 10. The figures only show envelope spectra at the frequency band from 0 Hz to 800 Hz, and harmonics of *f_I_* are flagged with vertical red dashed lines for a clear view. Figure 10a shows the signal measured at Point 1. Repetitive transient impulses are obvious in the time domain, and so are the fault-related impulses in the envelope spectrum. Figure 10b presents the signal measured at Point 2. The impulsive phenomena are not obvious in the time domain due to the contamination of noise. The envelope spectrum is also masked by noise, in which only two harmonics with small amplitudes can be seen. As shown in Figure 10c, the envelope spectrum of the signal measured at Point 3 cannot even identify any harmonic of *f_I_*. Therefore, signals measured at Point 1, Point 2 and Point 3 are in situations with low interference, medium interference and heavy interference, respectively.

**Figure 11:**
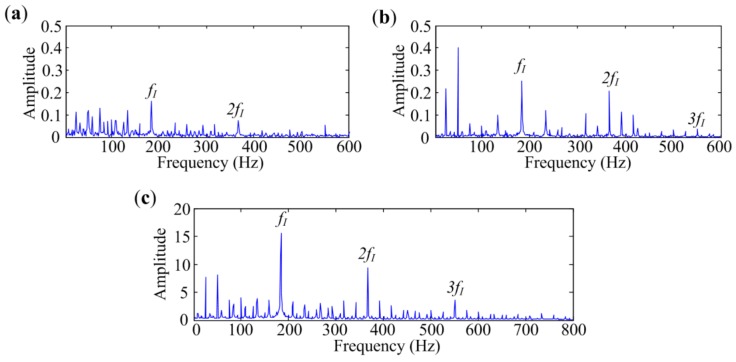
Analyzed results of signal with inner-race defect measured at Point 1 by different methods: (**a**) The fast Kurtogram; (**b**) Protrugram; (**c**) MESE.

**Figure 12:**
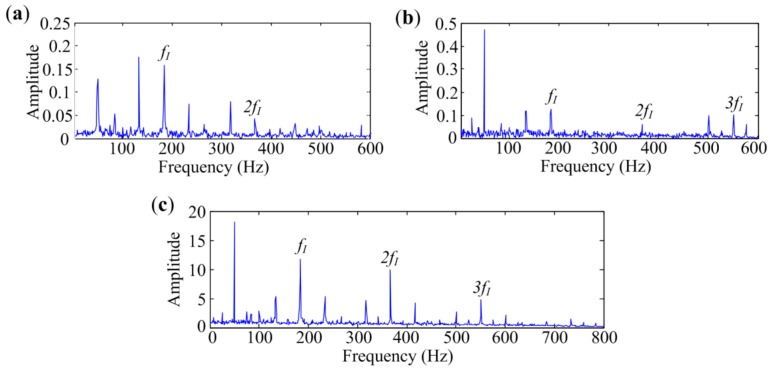
Analyzed results of signal with inner-race defect measured at Point 2 by different methods: (**a**) The fast Kurtogram; (**b**) Protrugram; (**c**) MESE.

**Figure 13:**
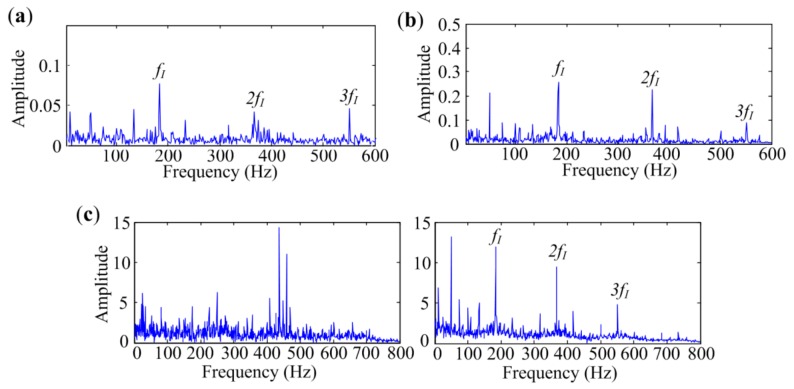
Analyzed results of signal with inner-race defect measured at Point 3 by different methods: (**a**) The fast Kurtogram; (**b**) Protrugram; (**c**) MESE.

To verify the effectiveness of MESE in bearing defect identification, Protrugram [17] and the fast Kurtogram [13] are introduced to analyze the same signals for comparisons. The envelope spectra extracted by different methods are presented in Figure 11, Figure 12 and Figure 13. As mentioned in [17], the bandwidth for Protrugram is set to 600 Hz, which is slightly more than three times the sought characteristic frequency, and the bandwidth of MESE is set to 800 Hz, to detect more harmonics.

Figure 11a shows envelope spectrum of the signal measured at Point 1 extracted by the fast Kurtogram. The narrowband envelope spectrum demodulated by Protrugram is presented in Figure 11b, where the rotating frequency component has the biggest amplitude. Envelope spectrum extracted by MESE is displayed in Figure 11c, in which the in-band noise is suppressed, and sideband frequency components are obviously enhanced. MESE has the best performance in this case. All three methods can figure out the characteristic frequencies, as the bearing is severely damaged, and the measuring point is close to the bearing.

Figure 12 shows the analyzed results of the signal measured at Point 2. As exhibited in Figure 10b, traditional envelope analysis has a weak performance in extracting fault features from this signal. Figure 12a is the spectrum obtained by the fast Kurtogram. The analyzed result of Protrugram is presented in Figure 12b. Although these two enhanced methods succeed in identifying the characteristic frequencies, the amplitudes are small and the harmonics are not protruding in the spectra. Envelope spectrum achieved by MESE is provided in Figure 12c. As seen in the spectrum, the noise frequency components are reduced and the harmonics of *f_I_* are significantly enhanced, which can strengthen the visual inspection ability. Although the three methods mentioned above are effective in detecting the bearing inner-race defect in the experiment, spectrum obtained by MESE is the clearest to show the inner-race fault characteristic frequency and its harmonics.

Figure 13 shows the analyzed results of the signal measured at Point 3. Figure 13a is the spectrum obtained by the fast Kurtogram. Harmonics are not protruding in the spectrum. The analyzed result of Protrugram is presented in Figure 13b. As seen in the spectrum, fault-related frequency parts are significantly enhanced. That means Protrugam has a better performance than the fast Kurtogram for inner-race fault diagnosis in situations with heavy interference. The envelope spectrum achieved by the proposed method is provided in Figure 13c. Two independent source spectra are extracted, and the fault features appear in the 2nd spectrum. The proposed method almost has the same performance as Protrugram, by which the visual inspection ability is significantly enhanced. For signals with inner-race defect measured at the three points, MESE is superior to the other two methods in in-band noise suppression and characteristic frequency components enhancement. So it performs better than the fast Kurtogram and Protrugram in bearing inner-race defect identification.

### 4.2. Bearing Outer-Race Defect Identification

**Figure 14:**
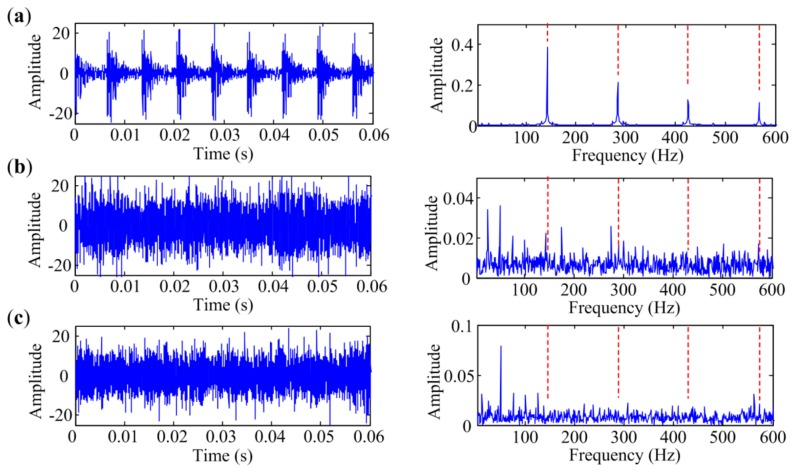
Bearing vibration signals measured at different points with outer-race defect: (**a**) Point 1; (**b**) Point 2; (**c**) Point 3.

For this test, the rotational frequency *f_R_* is 25 Hz and the characteristic frequency of outer-race defect *f_O_* is 140 Hz. We also measured vibration signals at Point 1, Point 2 and Point 3 using accelerometers. The time signals and corresponding envelope spectra are displayed in Figure 14. Envelope spectra at frequency bands from 0 Hz to 600 Hz are presented in the figures. The traditional envelope analysis can easily extract fault features from the signal measured at Point 1, as shown in Figure 14a. However, the envelope spectra of signals measured at Point 2 and Point 3 are contaminated by noise and do not show any fault characteristic components, as illustrated in Figure 14b,c.

**Figure 15:**
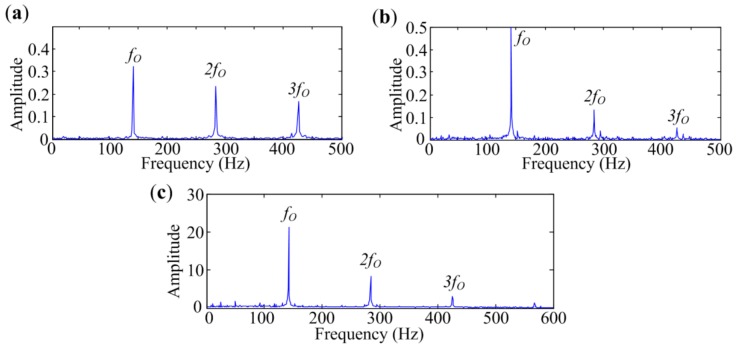
Analyzed results of signal with outer-race defect measured at Point 1 by different methods: (**a**) The fast Kurtogram; (**b**) Protrugram; (**c**) MESE.

The fast Kurtogram and Protrugram are also introduced to analyze the signals for comparisons. Bandwidth of Protrugram is set to 500 Hz, which is slightly more than three times the sought characteristic frequency. The bandwidth for MESE is chosen to be 600 Hz. Analyzed results of the signal measured at Point 1 are presented in Figure 15. Outer-race defect features extracted by the three methods are the same as that extracted by the traditional envelope analysis.

**Figure 16:**
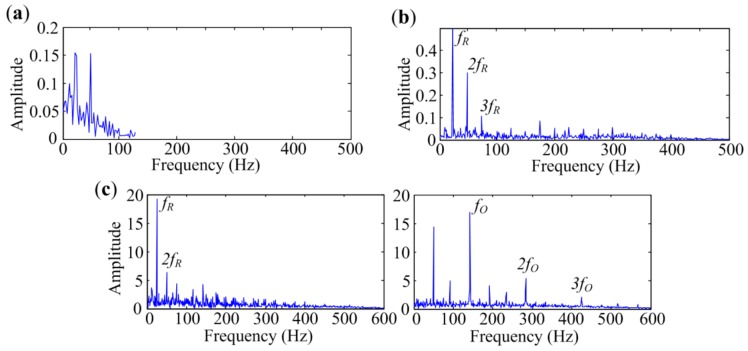
Analyzed results of signal with outer-race defect measured at Point 2 by different methods: (**a**) The fast Kurtogram; (**b**) Protrugram; (**c**) MESE.

Analyzed results of signal measured at Point 2 are presented in Figure 16. It can be seen in Figure 16a,b that the fast Kurtogram and Protrugram both fail to detect the bearing fault as the *f_O_* components cannot be found in the spectra. The envelope spectrum achieved by Protrugram is dominated by rotational components *f_R_*, which may be the main interference in the signal. Two independent source spectra are obtained by the MESE method, as exhibited in Figure 16c. The 1st spectrum contains lines at shaft rotational frequency *f_R_* and the main components of the spectra lines in the 2nd spectrum are the outer-race characteristic frequency *f_O_* and its harmonics. Since the fault characteristic components are clearly identified, the method can successfully detect the defect in bearing outer race. It also can be concluded from the results that the main interferences affecting bearing fault diagnosis are rotational components in the signal.

**Figure 17:**
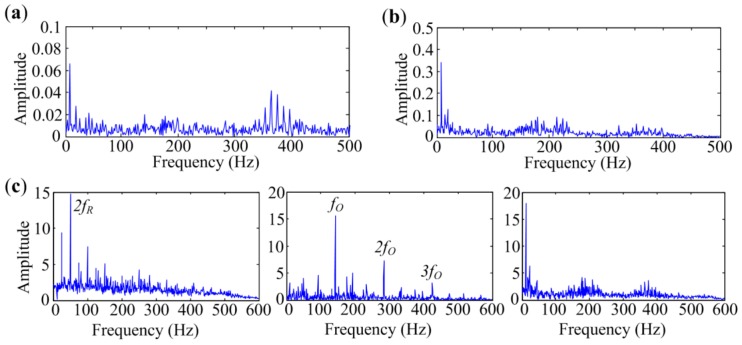
Analyzed results of signal with outer-race defect measured at Point 3 by different methods: (**a**) The fast Kurtogram; (**b**) Protrugram; (**c**) MESE.

The analyzed results of the signal measured at Point 3 are presented in Figure 17. As shown in Figure 17a,b, the fast Kurtogram and Protrugram also fail to detect the bearing fault, as the *f_O_* components are not evident in the spectra. MESE extracts three independent source spectra, as shown in Figure 17c. The first spectrum is dominated by rotational components, and the second one is a fault-related spectrum. The last spectrum contains the frequency 11 Hz and its harmonics, which may be the motor-related interference, as the measuring point is near the induction motor. It can be seen in the fault-related spectrum that the outer-race fault characteristic frequency *f_O_* and its harmonics are clearly identified, which means MESE has outstanding performance in outer-race defect identification. All the results in this subsection indicate that the proposed method has an enhanced performance in outer-race fault diagnosis even in heavy interference situations.

In this section, several real bearing fault signals are analyzed by the proposed MESE method, the fast Kurtogram method and the Protrugram method. These signals are measured at different points along the shaft to simulate situations with varying degrees of interference. For the signals measured near the tested bearing, although all three methods can successfully detect the two different kinds of bearing faults, MESE has the best visual inspection ability. When signals are measured far from the tested bearing, the fast Kurtogram and Protrugram have weak performance in fault diagnosis. In the case of outer-race defect, these two methods even fail to provide any bearing fault signatures in the extracted envelope spectra. However, the envelope spectrum obtained by MESE could clearly show the fault characteristic frequency and its harmonics both in the inner-race defect and outer-race defect situations. After the comprehensive comparisons were done, MESE showed the best performance among the three methods for bearing defect identification in our case studies. Therefore, the proposed method is effective and robust in bearing fault diagnosis.

## 5. Conclusions

In narrowband demodulation, criteria used to determine the optimal frequency band are easily affected by some other interference components in the vibration signal. Therefore, selecting signal just in one frequency band may lead to inaccurate results in extracting the fault transient components. To overcome these shortcomings, this paper proposes a method of multiband envelope spectra extraction for enhanced bearing fault diagnosis. This method is inspired by the characteristics of the repetitive transient impulses presented in the bearing fault signal: (1) repetitive transients are present as impulses in envelope spectrum, which can lead to high values of criteria; (2) repetitive transients are spread over a wide frequency range, so the fault components appear in multiple narrow bands. The method is implemented by fusing the envelope spectra at multiple narrow frequency bands. These informative frequency bands are determined by Jarque-Bera statistics of envelope spectrum amplitudes. Fast independent component analysis is introduced to extract the fault-related source spectra in the selected bands. Since all impulse features are preserved, the diagnosis is more robust and accurate. Comparative studies on the simulated and real bearing signals indicate that the proposed method outperforms the fast Kurtogram and Protrugram in fault diagnosis. The experimental results show that the proposed method is able to extract fault-related signatures from signals with heavy interferences and is an effective and reliable method for rolling element bearing faults diagnosis.

## Figures and Tables

**Table 1:** Parameters used for REB fault signal simulation. (*f_s_* is sampling frequency).

Parameters	*f_s_* (Hz)	*f_r_* (Hz)	*A_j_*	*Β*	*T* (s)	SNR (dB)
values	65,536	3800	1.0	1000	0.005	−20

**Table 2:** Parameters of bearings. (*f_R_* is the shaft frequency).

Type	Inside Diameter	Outside Diameter	Ball Diameter	Number of Balls	BPFI *f_I_* (Hz)	BPFO *f_O_* (Hz)
6010	50	80	9	13	7.4*f_R_*	5.6*f_R_*
